# Delivering Elder- and Community-Led Aboriginal Early Childhood Development Research: Lessons from the Ngulluk Koolunga Ngulluk Koort Project

**DOI:** 10.3390/children6100106

**Published:** 2019-10-01

**Authors:** Brad M. Farrant, Carrington C. J. Shepherd, Carol Michie, Clair Scrine, Michael Wright, Nicole Ilich, Tanya Jones, Glenn Pearson

**Affiliations:** 1 Telethon Kids Institute, The University of Western Australia, P.O. Box 855, West Perth, WA 6872, Australia; Carrington.Shepherd@telethonkids.org.au (C.C.J.S.); Carol.Michie@telethonkids.org.au (C.M.); Clair.Scrine@telethonkids.org.au (C.S.); Nicole.Ilich@telethonkids.org.au (N.I.); Tanya.Jones@telethonkids.org.au (T.J.); Glenn.Pearson@telethonkids.org.au (G.P.); 2 Ngangk Yira Research Centre for Aboriginal Health & Social Equity, Murdoch University, Perth, WA 6150, Australia; 3 School of Occupational Therapy, Social Work and Speech Pathology, Curtin University, P.O. Box U1987, Perth, WA 6845, Australia; M.Wright@curtin.edu.au

**Keywords:** elder-led research, community-led research, participatory action research, aboriginal early childhood development, Indigenous, first nations

## Abstract

Elder- and community-led research processes are increasingly being acknowledged as critical for successful Aboriginal health and wellbeing research. This article provides an overview of the methodologies, methods and progress of the Ngulluk Koolunga Ngulluk Koort (Our Children, Our Heart) project—an Elder- and community-led research and research-translation project focused on the early childhood development of Australian Aboriginal children in an urban context (Perth, Western Australia). We describe the application of a participatory action research methodology that is grounded in Aboriginal worldview(s), from the collaborative development of the original idea to the post-funding processes of co-design and implementation, data collection, analysis, interpretation and translation.

## 1. Introduction

The Ngulluk Koolunga Ngulluk Koort (Our Children Our Heart) project originated from numerous conversations with Aboriginal parents, Elders and researchers over the course of a number of years beginning in 2010. These conversations around early childhood development focused on the need for research to take the implications of differences in worldviews, values, language and culture seriously. At the time, Brad—a Wadjella (non-Indigenous person)—was completing a PhD in early childhood development and beginning a post-doctoral fellowship at the Institute for Child Health Research (now Telethon Kids Institute). What had become abundantly clear was that continuing to research and document the early childhood development of largely middle-class children from mainstream Western society was not helping to address the disadvantage that many children face but was instead largely providing further information to the parents of already advantaged children.

Many of the earliest conversations were with a Noongar Elder and researcher, often during cultural learning journeys on Noongar Boodjar (country). Soon after Brad started work at the Institute, he began discussing these issues with Glenn Pearson (Noongar man and Head of Aboriginal Research) who then brought Noongar researchers Prof Cheryl Kickett-Tucker and Dr Michael Wright into the conversation.

## 2. Background

The backdrop for these conversations remains the ongoing disadvantage that many Aboriginal Koolunga (‘children’ in Noongar language) and families face and the gap in life outcomes between Aboriginal and non-Aboriginal Australians, which have significant economic, social and personal costs that compel us to respond. The project is located in metropolitan Perth, which covers an area of approximately 6418 square kilometres and spans the majority of the Boodja (land) of the Whadjuk people. The Whadjuk people are one of 14 Noongar clans in the south-west of Western Australia. The 30,000 people of the Noongar nation form the biggest Aboriginal nation in Australia. While the project is situated on Noongar Whadjuk Boodja, it is worth noting that over one-third (38.7%) of Western Australia’s Aboriginal population live in Perth [1] and that many of them do not identify as Noongar.

Aboriginal people have occupied mainland Australia for at least 65,000 years, predating the human settlement of Europe and the Americas [2,3]. During this long period of connection to the land, Aboriginal people developed highly sophisticated understandings (science) and wisdom (e.g., living in harmony with the land in sustainable ways) that are reflected in culture through story, song, dance and art [4]. These have survived the processes of colonisation and, supported by an emphasis on rejuvenation and learning from Elders, continue to underpin the worldview(s) of Aboriginal people, including those who live on Noongar Whadjuk Boodja. This is a testament to the inherent strengths of Aboriginal people and culture.

The colonisation of Australia by the British began in what is now known as Sydney, New South Wales, in 1788, whereas European settlement began on Whadjuk Boodja (Perth) in 1829 with the establishment of the Swan River Colony. Colonisation has involved the wholesale theft of land (under the now discredited doctrine of *Terra Nullius*), massacres of Aboriginal people and other atrocities, the removal of Aboriginal people from their families and placement in missions (referred to as the *Stolen Generation*(s)), imprisonment, stolen wages, racism, denial of access to traditional languages, culture, and ways of being as well as other forms of oppression [5]. The systemisation and institutionalisation of discrimination and oppression has been a central, pervasive feature of colonisation, extending to wider government policies of control, exclusion and segregation [6]. These injustices have caused great harm to Aboriginal people, families, communities, culture and language that persist across generations to the present day, impacting on all dimensions of the holistic notion of Aboriginal wellbeing [7].

The purpose of the Ngulluk Koolunga Ngulluk Koort project is to help ameliorate the disadvantage that many Aboriginal Koolunga and their families face, by building on the strengths of Aboriginal people and culture. The focus on early childhood development, derived from the findings of international and national research, suggests that differences in early childhood development play a major role in the disparities in life outcomes experienced by Aboriginal Australians [8,9,10,11,12,13,14]. Findings from the Australian version of the Early Development Index, the Australian Early Development Census (AEDC—a mainstream, validated composite index of child development upon transition to formal schooling), indicate that there are significant gaps between the early childhood development and “school readiness” of Aboriginal and non-Aboriginal Koolunga. These and other findings have been incorporated into a deficit discourse [15] that often fails to recognise or acknowledge that these measures assess Koolunga against a set of understandings that are derived from a mainstream Western framework. This discourse is blind to the fact that the values, capacities and expectations that underpin measures like the AEDC are likely to be significantly different to those held by Aboriginal people. Difference does not equal deficit.

Measures of school readiness should be as much about the school being ready for the child as it is about the child being ready for the school [16]. Similarly, it is important that all early childhood development policy and practice is ready/appropriate for all Koolunga, regardless of their ethnic and/or socio-economic background [17]. This is easier said than done. Indeed, the current focus of school readiness is primarily fixed on children being ready for the school, which contributes to ongoing assimilation pressures, the framing of difference as deficit, and the undermining of Aboriginal values and culture.

Early childhood development researchers as well as policy developers need to recognise that failing to understand differences in culture and values can jeopardise the ethics and quality of their research, cause harm and undermine trust, and lead to policy failure [18,19]. Indeed, the Council for Aboriginal Reconciliation [20] argued that, while many acknowledge that education is vital for achieving reconciliation, this must go beyond the young people themselves to include the education of policy makers, decision makers, service providers, as well as the wider Australian community. The Closing the Gap Clearing House [21] investigated the principles and practices that underpin success in the area of early childhood development and found that these include: community involvement and engagement in both development and delivery; flexibility in design and delivery; continuity and coordination of programs; and the importance of building trust and relationships.

The Aboriginal colleagues and communities we work with have critically important expectations and values about the nature of the research they participate in. In order to effectively address inequality in early childhood development, we need a culturally appropriate fit between the values, needs and expectations of Aboriginal parents, Koolunga and families, and the resources and services that are available to them. However, Aboriginal parents and other community members highlight the relative absence of Aboriginal worldview(s) across mainstream early childhood approaches, resources and services and that this results in limited access by Aboriginal families and in reduced opportunities to improve developmental outcomes.

To make significant progress in closing the gap in outcomes for Aboriginal and non-Aboriginal Australian children and their families, a new decolonised paradigm is required [22]: one that reframes early childhood service provision from an Aboriginal worldview; one that focuses on relationships and recognises the “importance of family, community and connection to country as places that provide sustenance, a sense of identity and meaning” [23]. The existing paradigm often disenfranchises and excludes Aboriginal people and denies the legitimacy of Aboriginal worldviews. We must recognise the importance and legitimacy of Aboriginal knowledge systems, including the ontological worldview (ways of knowing) and epistemological worldview (ways of doing) [22]. Good intentions are not enough; trust is the key, and building trust takes time; assumptions and preconceptions must be put aside, and a modest and respectful approach needs to be taken [22]. It is only through prioritising and deepening relationships, transparency and inclusiveness, regular feedback, deep listening, and learning that healing and empowerment can occur for Aboriginal peoples, in conjunction with the needed transformation of structures, policies and processes that undermine Aboriginal perspectives [22].

Complex issues require deep engagement and comprehensive deliberation [22]. The deleterious and disempowering effects of colonisation are often maintained by systems, processes and practices rather than by individuals. Decolonisation is a process rather than an outcome [22]. It includes the previously silenced voices in an ongoing discussion between those impacted by colonisation and those that benefit from colonialist practices [22]. This approach is focused on making the research space as safe as possible and empowering for Aboriginal people, by ensuring all aspects of the research are underpinned and guided by Aboriginal knowledge, practices, beliefs and values [24]. As such, an Aboriginal research methodology is used so that the research does not further oppress or marginalise [22]. The aboriginal research methodology is distinguished from other methodologies by the notion that knowledge is created and shaped, not by individuals alone, but through relationships with other people and the world around us [25]. In accordance with this belief, it is required that a close and transparent working relationship is established with the local Aboriginal community and maintained throughout all stages of the research project [24].

Within an Aboriginal research paradigm, the role of the researcher is not primarily focused on the individual pursuit of new knowledge. Rather the priority is to build respectful relationships and fulfil the inherent obligations which arise from forming these relationships with Aboriginal people and communities [25]. In turn, this viewpoint shapes how the research is designed, conducted and interpreted. Knowledge gained is not perceived to be owned by any one individual; it is shared with all [25]. In this way, research is a form of intervention that can be transformative for both participants and researchers [23]. In order to recognise and respect Aboriginal culture while simultaneously addressing the disadvantage that many Aboriginal Koolunga face, we need to make research work that empowers an Aboriginal perspective of the factors that promote strong early childhood development. We need to work together to close the gap between the lived experiences of Aboriginal Koolunga and their families and the early childhood policies, practices and resources whose defining purpose is to enable all of our Koolunga to fulfil their potential. To facilitate this, we need more early childhood development research that recognises cultural differences, is grounded in Aboriginal cultural values and is shaped, evaluated and translated by Aboriginal people.

We recognise that Aboriginal peoples have a holistic view of health that goes beyond individual physical and mental wellbeing to include aspects of culture, spirituality, language, connection to land, and the social, emotional, and cultural wellbeing of family and community. Indigenous and Western worldviews differ in a number of important ways. The holistic focus of indigenous worldviews (“everything and everyone is related”) contrasts starkly with the reductionism of Western worldviews (compartmentalisation/reduction into ever smaller parts) [26,27,28]. This has critical implications for how issues are seen and where the origins of problems are perceived. The reductive nature of Western science and the failure to engage with and understand holistic Aboriginal worldviews have been major barriers to the development of a coordinated, integrated and holistic research agenda and approach that is culturally informed. Aboriginal and Western worldviews also differ starkly on the origins of authority. While authority in Western systems is given through roles and bureaucracy, authority in Aboriginal communities is based on age, cultural knowledge and relationships [27]. In Noongar culture, Elders are the Boordiyas (Bosses). The process of decolonising research includes recognising and honouring the role and status of Elders in Aboriginal culture and putting them at the centre of the research process. As one of the participants in the Ngulluk Koolunga Ngulluk Koort project told us “Culture is connected to learning to respect your Elders, connection to the bush, being proud of our ancestors and proud of who we were, and then rebuilding yourself for the new, contemporary way to be a Noongar” [29].

The purpose of the Ngulluk Koolunga Ngulluk Koort project is to work with the urban Aboriginal population of Perth (predominantly comprised of the Noongar language group) to achieve these outcomes. The project includes Aboriginal community involvement and control via a participatory action research methodology, incorporating an Aboriginal worldview and knowledge framework developed by Noongar researcher Dr Michael Wright. Wright’s previous research (the Looking Forward project) demonstrated that this participatory action research approach is a highly effective tool that enables Aboriginal requirements to be met (including fulfilling the key principles of the Indigenous Research Reform Agenda), while delivering on a range of specific research outcomes. Rather than being performed on them, participatory research is carried out with and by the community (they have the control) and involves systematic inquiry, reflection and action [30,31]. It includes addressing power imbalances, mutual benefit among partners, and reciprocal knowledge translation [32]. It requires ongoing conversations/yarning to ensure that the interpretation of findings is understood and supported by all parties. This bottom-up participatory approach embodies the six core values: of reciprocity, respect, equality, responsibility, survival and protection, and spirit and integrity [33], and it is embraced by the Aboriginal community. It was from this perspective that what is now known as the Ngulluk Koolunga Ngulluk Koort project was first proposed.

## 3. Methodology

### 3.1. Proposed Research Methodology

The very nature of the control and responsivity of participatory action research means that the research plan is constantly in flux. At the time the grant application was submitted, the plan was to use a mixed methods approach in an ongoing reflective process (where the qualitative research informs and directs the quantitative research and vice versa), including the participatory action research methodology in an iterative fashion, to:

- Engage with groups of Aboriginal/Noongar Elders (Community Advisory Groups) in the Midland/Swan, Fremantle and Armadale/South East regions of Perth in order to build a deeper understanding of Aboriginal cultural values, customs and laws, as these relate to parenting practices and other processes that play important roles in early childhood development. This engagement with Elders has been critical to the success of the Looking Forward project and its successor the Looking Forward Moving Forward project—once the Elders trust the researchers, they ‘vouch’ for the researchers and the research project to the wider community and help to drive the reciprocal participatory action research process.
- Consult and collaborate with Aboriginal/Noongar parents, families and communities (Community Forums) in the Midland/Swan, Fremantle and Armadale/South East regions of Perth in order to delineate a community perspective of “what it takes to grow children up strong”.
- Discuss key early childhood development outcomes with the Aboriginal Community Advisory Groups, Aboriginal Cultural Consultants and the wider Aboriginal/Noongar community as well as the factors that have been found to prompt (e.g., parent-child socio-emotional engagement), facilitate (e.g., the home learning environment) and constrain (e.g., maladaptive parenting) these.
- Take the Aboriginal/Noongar worldview/perspective of “what it takes to grow children up strong” and use a strengths-based approach to compare and contrast this with the non-Aboriginal/Western perspective of early childhood development. This will result in the development of research questions and associated hypotheses that have the capacity to increase our understanding and ability to close the gap in early childhood development.
- Quantitatively assess these research questions and hypotheses via a secondary analysis of data from three large existing surveys (the Longitudinal Study of Indigenous Children, the Western Australian Aboriginal Child Health Survey, and the Longitudinal Study of Australian Children).
- Discuss and refine the results of the quantitative analyses with the Elders (Community Advisory Groups) and then with parents and the broader Aboriginal community (Community Forums).
- Working with the Aboriginal/Noongar communities as well as policy makers, government agencies, service providers, and other stakeholders (Policy and Service Provider Reference Group), use a consultative and collaborative process to expand everyone’s understanding of early childhood development and produce a suite of culturally appropriate and empowering policies and practices that connect early childhood development outcomes to the strengths of Aboriginal/Noongar culture.
- Organise Community Gatherings to further disseminate and promote the research findings to the broader Perth Aboriginal/Noongar community.
- Engage with a broad range of policy makers, government agencies, service providers, and other stakeholders in a way that increases their understanding of the issues and facilitates the uptake of the resultant policies and practices.

The beginning of the proposed process focused on trust and relationship building, and inviting participants (Elders, community members, policy makers, service providers) to be involved in the design of the study [24], including the setting of research priorities, specification of the ethical design, the actual execution and evaluation of the research along with control of the ongoing surveillance, as well as the transformation and translation of the research findings into policy and practice. The decision to recruit Elders as co-researchers was based on the Looking Forward project [34]. The Elders/co-researchers bring their cultural knowledge, wisdom, professional skills and expertise to control and direct the research and ensure that the project is relevant to the local Aboriginal community’s needs, is rigorous, culturally safe, and aligns with the strengths of Aboriginal culture. The use of a participatory action research approach to inform and direct the quantitative research of existing data allows two very different methodologies with different strengths to complement each other. Research that identifies the similarities and the differences in the factors that prompt, facilitate and constrain early childhood development across cultures can potentially inform and drive changes in the way systems and services respond to Aboriginal children and families. It can also provide evidence which supports and validates Aboriginal families and communities as they pursue the goal of “growing their children up strong”.

### 3.2. Evolution of the Research Methodology

Work on the project began in January 2016, with Michael Wright providing input and guidance around methodology and engaging with Elders as co-researchers. A meeting was held with the Elders/co-researchers of the Looking Forward project in early April 2016 to seek their input and advice. The Elders advised that the new project should cover the whole of the Perth metropolitan area (rather than 3 sub-regions as had been proposed in the grant application). While this was a substantial expansion of the scope of the original project, it was supported by the research team in order to uphold the relational obligation to these Elders.

After discussions with an Aboriginal consultant, it was decided that the best way to move forward would be to hold a meeting of a relatively large group of Elders (approx. 25–30) from across Perth to seek support for the project and to call for volunteers to join the project as Elders/co-researchers. A meeting attended by 51 Elders from across the Perth metropolitan area was held in May 2016. At this meeting, an overview of the project including its goals and expected outcomes was provided, key members of the (then) research team were introduced, suggestions for a suitable name for the project were called for, the role of the Elder/co-researcher group was explained, and expressions of interest for joining the group were sought. The Elders present at this meeting provided unanimous support for the project and agreed that the metro area could be approached as four regions: South East (Belmont to Armadale), North East (Midland/Swan Area), North West (Perth City to Two Rocks), and South West (Fremantle to Rockingham). It was also agreed that a male and female Elder (where possible) should be included from each region. The expressions of interest that were received were supplemented as necessary with direct approaches to other Elders, in part to increase the number of male Elders. Nine Elders commenced as co-researchers on the project in June 2016. The membership of the Elder/co-Researcher group has been very stable with only one change (due to personal commitments) over the life of the project. The rest of the Elder/co-researcher group discussed how to fill this vacancy and did so by approaching individuals who were known to the community in the relevant region.

From the outset, the Elders/co-researchers have had input into almost every research decision. In initial group meetings, the co-researcher role was discussed, and expectations for this role and the research team’s role were clarified and agreed upon. A Terms of Reference for the group was drafted, revised in response to feedback and endorsed by all members after much consideration. The guiding vision statement included in the Terms of Reference includes direct quotes provided by the Elders. Group members have provided advice on project staff recruitment and identified community organisations and government departments they wanted to involve in the project. Some of the Elders/co-researchers have also sat on research staff recruitment selection panels. As a result of the diverse range of perspectives existing within the Elder/co-researcher group, it can take some time to reach consensus among the group, but enabling the decision-making process to unfold naturally corresponds with Aboriginal epistemology and ontology.

For example, the process of settling on a name for the project required considerable group deliberation over several meetings, as well as input from each individual in between meetings. After ruling out names that were already in use or had been used previously, as well as those that were likely to be confused with other names, the Elder/co-researcher group selected the name of the project from a list of suggested Noongar names. The development of the community engagement strategy was also guided by the Elder/co-researcher group. The wider Aboriginal community of Perth was engaged via a series of community forums and focus groups that were co-facilitated by the Elders/co-researchers across the four regions. All participants gave their informed consent for inclusion before they participated in the research. The research was conducted in accordance with the Declaration of Helsinki, and the protocol was approved by the Western Australian Aboriginal Health Ethics Committee (Reference number: 674) and the University of Western Australia’s Human Research Ethics Committee (Reference–RA/4/1/7963). Participants for the community forums and focus groups were recruited via the project team and Elders’/co-researchers’ networks, including community-based organisations. Participants ranged in age from 18 to over 80 years. The four community forums (one in each region) were attended by 80 participants who were largely senior members of the Aboriginal community. To ensure the input of younger people, the Elders/co-researchers proposed that focus groups be held at venues attended by younger parents. Seven focus groups were held across the four regions, including a dedicated men’s group involving participants of mixed ages. The focus groups were designed to support younger members to share their views, and they attracted 58 participants. The community forums and focus groups were led by Aboriginal people, with an Aboriginal facilitator overseen by the Elders/co-researchers from the relevant region and supported by the project team.

The participants at each of the community forums and focus groups discussed the following questions, which were developed and piloted by the Elders/co-researchers to ensure that they were suitable:

1. What are the moorditj [good] things that are important in raising strong, solid young kids (under 6 years of age);
2. What things might get in the way of Aboriginal kids (under 6 years of age) growing up solid in Perth;
3. Thinking about kids under the age of 6 years, what things help Aboriginal kids grow up solid (resilient, confident, happy, healthy)?

The discussions at the community forums and focus groups were digitally recorded, transcribed, and a conventional thematic content analysis was applied.

## 4. Ngulluk Koolunga Ngulluk Koort Framework

**Figure 1:**
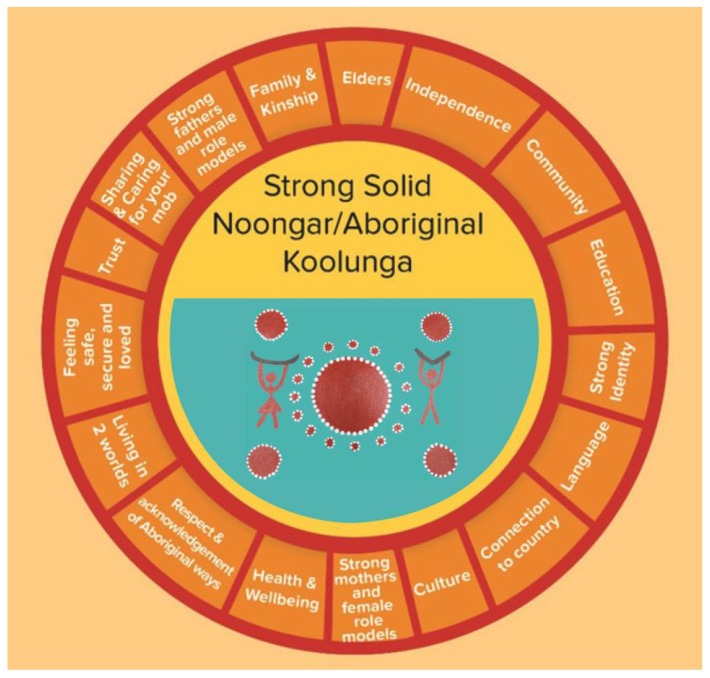
Acknowledging our ways of raising strong, solid Koolunga (Reprinted with permission from the Ngulluk Koolunga Ngulluk Koort project, Telethon Kids Institute).

**Figure 2:**
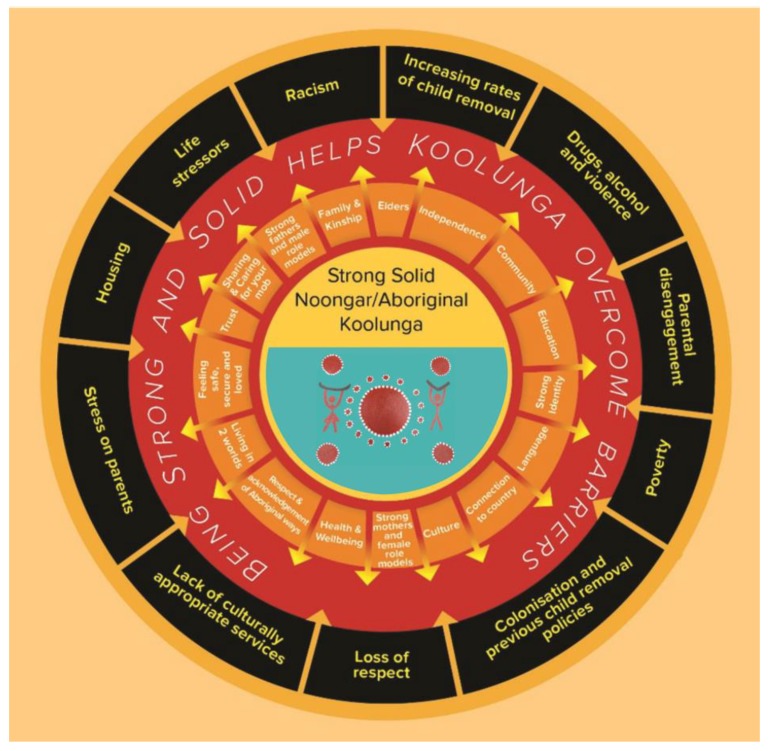
Things that get in the way of raising strong, solid Koolunga (Reprinted with permission from the Ngulluk Koolunga Ngulluk Koort project, Telethon Kids Institute).

From this process, the Ngulluk Koolunga Ngulluk Koort Framework emerged. This framework represents the factors that the Aboriginal people of Perth identified as critical for growing strong and solid Koolunga. The first diagram (Figure 1) is an important reflection of the strengths and positive values of Noongar/Aboriginal ways of raising kids. The second diagram (Figure 2) includes all the main issues people at the forums and focus groups raised as concerns that many Aboriginal families have to deal with, and which often stop children and their families from being strong and solid. In addition to producing the Ngulluk Koolunga Ngulluk Koort Framework, the community consultations highlighted 3 main priority areas of concern to the community. These are: (1) the impact of child removal; (2) the importance of education across the early years (early childhood education and care, and early schooling); and (3) the lack of housing security for many Aboriginal families.

The Ngulluk Koolunga Ngulluk Koort Framework received endorsement by the Elders at the second meeting of Elders from across the Perth metropolitan region. This meeting was held in October 2017 and was attended by 60 Elders who also endorsed what the project had done so far and emphasised the need to make sure that the project’s research findings are translated into changes to policies and services.

## 5. Research Translation Activities

**Figure 3:**
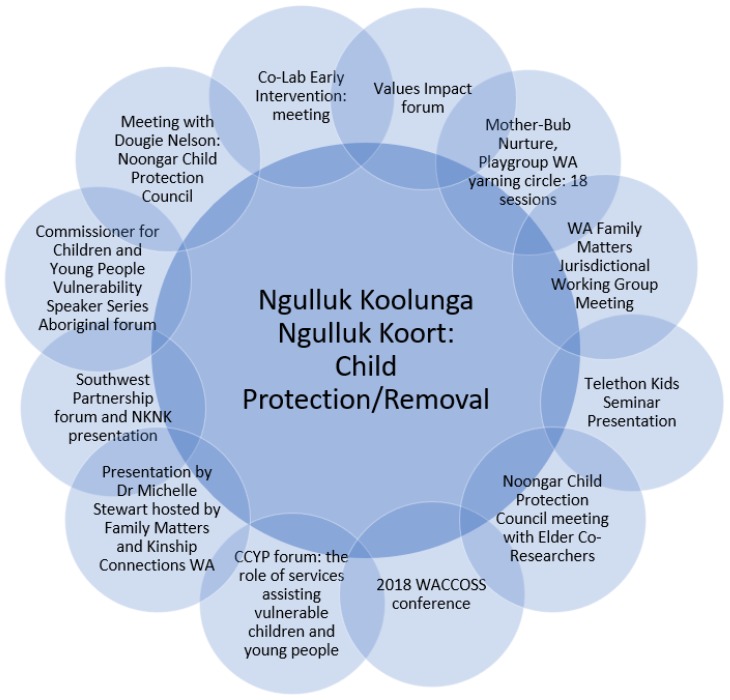
Child protection/removal research translation activities (Reprinted with permission from the Ngulluk Koolunga Ngulluk Koort project, Telethon Kids Institute).

The project’s focus throughout 2018 was to work with stakeholders to translate the research findings into changes to policy and practice. In relation to child protection/removal, our work has been directed at supporting Aboriginal community-controlled organisations (which the Elders/co-researchers are very keen to support wherever they can) working in this area, as well as participating in relevant consultation processes and forums. The Elders/co-researchers and project team want to ensure a unified voice among the community with regard to the issue of child protection/removal and ensure that Aboriginal-led solutions are appropriately funded and supported by government. In the child protection/removal space, the Ngulluk Koolunga Ngulluk Koort Elders/co-researchers and the project team have attended and participated in a broad range of activities (Figure 3).

**Figure 4:**
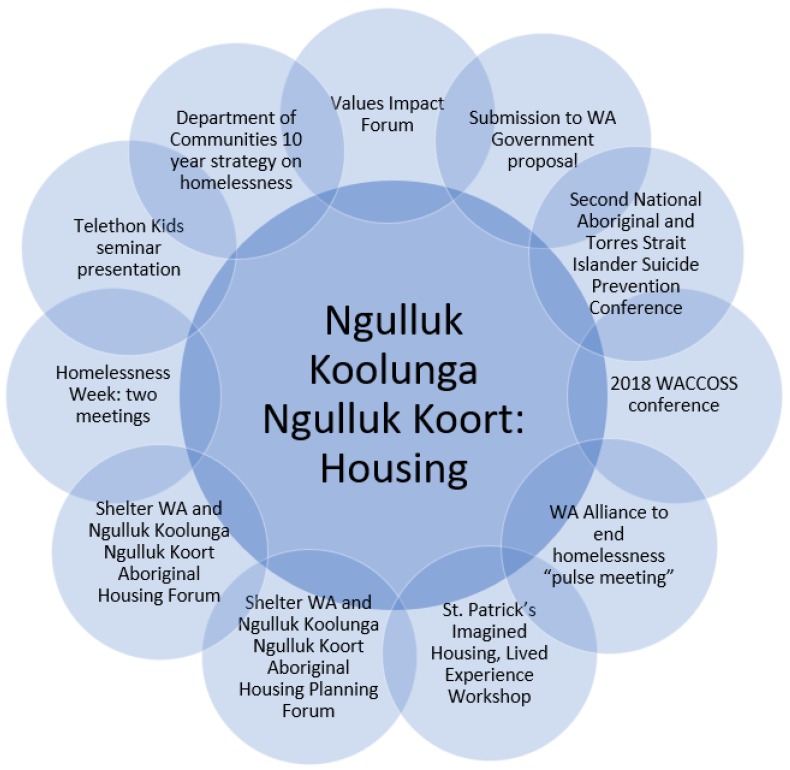
Housing and homelessness research translation activities (Reprinted with permission from the Ngulluk Koolunga Ngulluk Koort project, Telethon Kids Institute).

In relation to housing and homelessness, the team’s work has been directed at connecting the project with relevant housing organisations and advocacy groups, as well as participating in consultation processes and forums and contributing to the development and implementation of strategies. The Elders/co-researchers and project team have sought to ensure that issues that specifically impact Aboriginal children are given prominence and consideration, underlining the need for Aboriginal-led solutions. The project will continue to seek to build connections within the sector, identifying opportunities for advocating for change and to ensure the voices, values and priorities of Aboriginal children and their families are heard and considered. In relation to housing and homelessness, the Ngulluk Koolunga Ngulluk Koort Elders/co-researchers and the project team attended and participated in a broad range of activities, including those represented in Figure 4.

After participating in these activities and building/extending the necessary foundation of relationships, in August 2018, the Ngulluk Koolunga Ngulluk Koort project team arranged to meet with representatives from Shelter WA (the peak housing body) and a range of Aboriginal Community Controlled Organisations to discuss ways to address the number of Aboriginal people without a home and who do not have stable tenancy arrangements across metropolitan Perth. Issues that were raised included the supply of housing and expense of the private market; the poor maintenance of housing; the length of waitlists (5000 on a list for over 5 years); maintaining tenancies (evictions, 3 strike policy—current state government policies result in the eviction of government social housing tenants if 3 substantiated complaints/breaches occur); the effects of personal trauma that manifest in so many areas that impact on both housing and the ability to keep a house; and the lack of income security. This meeting laid the foundation for the Metropolitan Aboriginal Housing Forum, a forum jointly developed by Shelter WA and the Ngulluk Koolunga Ngulluk Koort project and held in November 2018. The forum included a number of presentations from different stakeholders (NGO and Government) associated with the issue of Aboriginal people’s experiences of housing and homelessness in metropolitan Perth. The next steps identified at the Metropolitan Aboriginal Housing Forum included: Shelter WA, in partnership with Telethon Kids Institute, to develop a working group with the Elders as a platform for an Aboriginal voice in Noongar housing policy and to develop an Aboriginal housing policy and framework to measure progress; Shelter WA to discuss the development of a Noongar Community Housing Strategy with Noongar Mia Mia (Aboriginal-controlled housing provider organisation) to build on the value proposition of Noongar-managed housing, to address the lack of safe, secure and culturally appropriate housing supply, and to capitalise on new opportunities; Shelter WA to develop, in partnership with Noongar Mia Mia, other agencies as appropriate and the Department of Communities, new models for affordable home ownership schemes, i.e., a rent-to-buy option with long-term rental payments to become shared equity in ownership; and Shelter WA to ensure Aboriginal people are central to the review of the Residential Tenancies Act 1987. Shelter WA was also urged to work in partnership with the Aboriginal community to increase advocacy for: the abolishment of the three strikes policy; Aboriginal advocates to support people and liaise with the Department of Communities on housing issues; the Department of Communities to simplify housing jargon; the Department of Communities to undertake a cultural audit of current government housing policies and practice; a review of the negative impact of employment outcomes on social housing eligibility and the development of affordable and secure housing transition options; a policy to ensure that safe and stable housing and support is provided immediately for people being released from government institutions [35].

The third meeting of Elders from across the Perth metropolitan region was held in February 2019. This meeting was attended by 65 Elders who heard presentations from the Noongar Family Safety and Wellbeing Council, Noongar Mia Mia, and Shelter WA. At this meeting, the Elders in principle gave support for the establishment of the Noongar Family Safety and Wellbeing Council and for the fact that the Elders/co-researchers would work with the Council to ensure the appropriate and effective cultural governance of the Council. The Elders endorsed the next steps from the Shelter WA and Ngulluk Koolunga Ngulluk Koort Metropolitan Aboriginal Housing Forum, as detailed above, and recognised Noongar Mia Mia as the peak housing body for Noongar and Aboriginal people in Perth. The Elders also endorsed the proposal that Elders/co-researchers work with Noongar Mia Mia to develop a Housing Standards Code of Conduct of what is expected for all tenants that occupy a Noongar Mia Mia property. Once developed, this will be included as part of the conditions of the Residential Tenancy Agreement that all tenants sign when taking a Noongar Mia Mia property. The Elders also agreed that Noongar Mia Mia meet with Elders quarterly or when practical to provide an update on the progress of housing matters as identified. The Ngulluk Koolunga Ngulluk Koort project team is facilitating these processes to ensure that these points are actioned and that change occurs.

## 6. Discussion/Next Steps

The Ngulluk Koolunga Ngulluk Koort project has demonstrated the power of Elder- and community-led research processes to help transform and decolonise research, policy and service provisions. Key learnings include developing better understandings of the implications of differences in worldviews, values and culture. In particular, it is critical that differences in the origins of authority are understood, honoured and respected. In the context of Noongar people and culture, this means engaging with and being led by Elders in each step of the research process, as well as including the voices of other community members (e.g., parents of young children). Research thus becomes both culturally appropriate and part of the decolonisation process. This approach is transferable to other colonised contexts and is a necessary step to ensuring that research contributes to liberation rather than ongoing oppression. This work consumes significant amounts of time and resources in order to build the necessary relationships and trust, address power imbalances, achieve consensus (as much as possible), and work with a range of stakeholders to translate the findings of the research to achieve real change to improve outcomes for children and families. This needs to be included in research planning, timelines and grant applications, and is a central aspect of governance structures.

We are committed to enabling and producing early childhood development research that lives up to the expectations and values of the Aboriginal colleagues and communities we work with. We have recently recruited a new staff member to the team, with the primary aim of using the Ngulluk Koolunga Ngulluk Koort Framework to inform and direct quantitative research using existing large datasets, to provide additional evidence as required for the Framework and its translation into policy and practice. An Aboriginal Health Promotion Association Scholarship holder is also working with the project this year to develop health promotion resources to support the parents and carers of Aboriginal children in engaging with early childhood education service providers to improve the cultural awareness, cultural safety and cultural security of early education settings. The ongoing use of participatory action research and the leadership of the Elder/co-researcher group (and the broader group of around 100 Elders that the project reports to) will ensure that the Aboriginal community will continue to control the direction and focus of the research so that the benefits gained are aligned with the community’s values and priorities.

Working with stakeholders, including Aboriginal community-controlled organisations, service providers and policy makers, to translate the research findings into changes to policies and services continues to be the major focus of our work in 2019. The goal is to ensure that the knowledge that is generated by the research is exchanged and translated between the Aboriginal community, researchers, service providers and policy makers. This ongoing engagement with early childhood development service providers and policy makers will continue to increase their capacity to deliver culturally safe and empowering policies and practices.

An inadequate depth and longevity of community engagement severely limits the effectiveness of much Aboriginal research. The goal is to work with the Elders and the broader Aboriginal community of Perth to expand Ngulluk Koolunga Ngulluk Koort into a program of family-centred Aboriginal early childhood development research. To support this, we are currently developing research grants where the research ideas and questions emerge from a conversation with the Aboriginal community; the research projects are co-designed with the community, and are enacted in partnership with the community. The overall vision is for a comprehensive program of Elder-led research comprised of a range of community driven and integrated research projects funded from a diverse range of sources and delivered in collaboration with a broad range of government and non-government service providers and policy makers. While this kind of community-led research, which involves engaging a large range of stakeholders in sometimes very difficult conversations, is necessarily high-risk/high-reward in nature, the evidence clearly indicates that this is what is needed to bring about the required transformative changes.
